# Electrically stimulated hind limb muscle contractions increase adult hippocampal astrogliogenesis but not neurogenesis or behavioral performance in male C57BL/6J mice

**DOI:** 10.1038/s41598-020-76356-z

**Published:** 2020-11-09

**Authors:** Jennie C. Gardner, Svyatoslav V. Dvoretskiy, Yanyu Yang, Sanjana Venkataraman, Dominica A. Lange, Shiping Li, Alexandria L. Boppart, Noah Kim, Catarina Rendeiro, Marni D. Boppart, Justin S. Rhodes

**Affiliations:** 1 Department of Psychology, University of Illinois at Urbana-Champaign, Champaign, USA; 2 Department of Kinesiology and Community Health, University of Illinois at Urbana Champaign, Champaign, USA; 3 Beckman Institute for Advanced Science and Technology, University of Illinois at Urbana-Champaign, 405 N. Mathews Ave., Urbana, IL 61801 USA; 4 Present Address: School of Sport, Exercise and Rehabilitation Sciences, University of Birmingham, Birmingham, UK

**Keywords:** Biophysics, Immunology

## Abstract

Regular exercise is crucial for maintaining cognitive health throughout life. Recent evidence suggests muscle contractions during exercise release factors into the blood which cross into the brain and stimulate adult hippocampal neurogenesis. However, no study has tested whether muscle contractions alone are sufficient to increase adult hippocampal neurogenesis and improve behavioral performance. Adult male, C57BL/6J mice were anesthetized and exposed to bilateral hind limb muscle contractions (both concentric and eccentric) via electrical stimulation (e-stim) of the sciatic nerve twice a week for 8 weeks. Each session lasted approximately 20 min and consisted of a total of 40 muscle contractions. The control group was treated similarly except without e-stim (sham). Acute neuronal activation of the dentate gyrus (DG) using cFos immunohistochemistry was measured as a negative control to confirm that the muscle contractions did not activate the hippocampus, and in agreement, no DG activation was observed. Relative to sham, e-stim training increased DG volume by approximately 10% and astrogliogenesis by 75%, but no difference in neurogenesis was detected and no improvement in behavioral performance was observed. E-stim also increased astrogliogenesis in CA1/CA2 hippocampal subfields but not in the cortex. Results demonstrate that muscle contractions alone, in absence of DG activation, are sufficient to increase adult hippocampal astrogliogenesis, but not neurogenesis or behavioral performance in mice.

## Introduction

Exercise is crucial for maintaining cognitive health throughout life^1^. Many neurological changes have been documented in response to exercise training in humans and rodent models, especially in the hippocampus^2,3^. One of the most robust of these adaptations is increased number of new neurons, along with astrocyte support cells, in the dentate gyrus (DG) of the hippocampus^4^, which results in increased volume of the granule cell layer of the DG in rodent models^5^. These new cells are incorporated into existing neuronal circuits, potentially enhancing hippocampus-related cognitive function, such as memory and learning^6,7^.

Despite the potential functional significance of newly generated neurons in cognitive performance, the mechanisms through which exercise enhances adult neurogenesis remain unclear. One current hypothesis is that factors released into circulation (e.g., lactate, myokines, peptides, growth factors) during muscle contraction, can cross into the brain to exert a neurotrophic influence^8^. Early studies investigated the role of growth factors, such as insulin like growth factor 1 (IGF-1) and vascular endothelial growth factor (VEGF), by specifically blocking their access to the brain after exercise and demonstrated that these factors were necessary for exercise-induced increases in adult hippocampal neurogenesis^9,10^. More recently, the myokine cathepsin B, which increases in circulation during exercise and can cross into the brain, was shown to be necessary for exercise-induced adult hippocampal neurogenesis and improved behavioral performance in rodents^11^. Other studies have also shown that muscle-derived factors, in particular lactate and fibronectin type III domain-containing protein 5 (FNDC5) and its cleaved product, irisin, can enhance expression of hippocampal brain derived neurotrophic factor (BDNF)^12,13^. Extensive evidence links BDNF to survival and proliferation of neurons in the hippocampus^14^.

In addition to the mounting evidence for myokine-driven neuroplasticity, there is also evidence showing that physical exercise induces acute neuronal activity in the hippocampus, which is strongly correlated with intensity of physical activity^15,16^. Importantly, neuronal activity alone can increase the expression of hippocampal BDNF^17^ and neurogenesis^18^, and possibly cathepsin B, IGF-1 and VEGF, which are all highly expressed in the hippocampus^19^. As such, the local expression of these and other factors driven by increased neuronal activity in the hippocampus during exercise could be crucial to induce neurogenesis in the hippocampus^3^.

Isolating the specific contribution of muscle contractions from hippocampal neuronal activation can be achieved by using an animal model in which muscle is contracted artificially during anesthesia. This strategy has already been used successfully to demonstrate that one trial of 50 repeated maximal isometric contractions of the triceps surae muscles of rats was sufficient to increase BDNF in the hippocampus^20^. However, whether chronic exposure to muscle contractions is sufficient to recapitulate the neuroanatomical and behavioral impacts of exercise in the brain in the absence of acute hippocampal activation remains unknown. The goal of this study was to determine the extent to which repeated electrically stimulated muscle contractions over a period of 8 weeks, in the absence of hippocampal activation, are sufficient to increase adult hippocampal neurogenesis and enhance behavioral performance in otherwise sedentary mice.

## Materials and methods

### Experimental subjects and general husbandry

Male, 3-month old, C57BL/6J mice were used (total n = 73) [Experiment 1, n = 32 (16/group); Experiment 2, n = 7 (4 sham and 3 e-stim); Experiment 3, n = 34 (17/group)]. Rooms were kept at a constant temperature of 72°F and set to a 12:12 light–dark cycle with lights on at 7:00 am and off at 7:00 pm. Food and water were provided ad libitum (Teklad Rodent Diet 2918). Corn cob bedding (Harlan Teklad 7097, Madison, Wisconsin, USA) was provided in all cages. All procedures were approved by the University of Illinois Institutional Animal Care and Use Committee and adhered to NIH guidelines.

### E-stim/Sham treatment

E-stim mice were subjected to bilateral electrical stimulation of the sciatic nerve as previously described^21^. Prior to treatment, mice were anesthetized using isoflurane at a concentration of 2–5% during induction. Anesthesia was maintained throughout the procedure via a nose cone and isoflurane was kept at a concentration from 1–3% throughout the procedure. Both hind limbs were then shaved and aseptically prepared before being placed one at a time in a miniature metal foot plate attached to the shaft of a servomotor (1300A, Aurora Scientific, Aurora, ON, Canada). The foot was placed so that it was perpendicular to the tibia. Two platinum electrodes were then inserted through the skin on either side of the sciatic nerve. A stimulator and stimulus unit were used to activate the sciatic nerve via the platinum electrodes to induce contraction of the crural muscles. We tested a range of frequencies prior to the experiment and settled on the optimal frequency 100 Hz (Hz) with pulses of 0.1 ms (ms) duration because of its high peak twitch force that was not further elevated by higher frequencies. Our voltage setting (100 mA @25%, ~ 1 V) elicited maximal force generation at 100 Hz, such that any increase above 1 V did not elicit any further increase in force. The plate then rotated during electrical stimulation so that the posterior (gastrocnemius, soleus) crural muscles contracted eccentrically (19° of ankle plantarflexion), forcing the anterior (tibialis anterior) crural muscles to contract concentrically. After 5 contractions, the plate rotated in the opposite direction during electrical stimulation so that the posterior crural muscles contracted concentrically (19° of ankle dorsiflexion), forcing the anterior crural muscles to contract eccentrically. Every set (5 eccentric, 5 concentric) was separated by 10 s rest periods. The stimulation protocol consisted of 4 sets, or a total of 40 contractions. Sham control mice were anesthetized and subjected to electrode insertion for a similar amount of time as the e-stim mice, but no electric current was delivered.

### Experiment 1: acute cFos and hippocampal cell proliferation study

The purpose of this experiment was to determine whether the muscle e-stim treatment acutely induces neuronal activation and cell proliferation in the DG. Two separate cohorts of mice were used (total n = 16/group). Mice were randomly assigned to either the electrical stimulation (e-stim) group or the sham group to serve as a control. One at a time, the mice underwent either a single e-stim session (approximately 15 min) or a single sham session. Sham and e-stim mice were alternated throughout the course of the experiment. After each mouse underwent either the sham or the e-stim condition, they were transported back to their holding room. Exactly 90 min post-e-stim/sham treatment, the mice were perfused with a 4% paraformaldehyde solution.

### Experiment 2: acute lactate study

The purpose of this experiment was to determine whether the muscle e-stim treatment acutely increases lactate in the blood serum, since muscle-derived lactate is a typical response to exercise of large muscle masses^22^, and because lactate was implicated in muscle-brain interactions in a previous study^13^. Immediately following the e-stim (n = 3) or sham (n = 4) procedure, the animals were euthanized and blood was taken via cardiac puncture. Serum was assayed using previously validated commercially available enzyme immunoassay kits for lactate (Abcam L-Lactate Assay Kit, ab65331)^23^. Each plasma sample was diluted 1:25 in assay buffer, prior to analysis following kit instructions. All samples were run in duplicate in a single assay. Subsequent absorbance was read using the Biotek Microplate Spectrophotometer (Biotek, Winooski, VT, USA) following manufactures’ instructions.

### Experiment 3: 8-week chronic e-stim study

The purpose of this experiment was to determine whether the chronic muscle e-stim treatment affected the cellular morphology of the hippocampus and behavioral performance of the mice. Experiment 3 consisted of 3 separate cohorts of mice (total n = 17/group). Mice were randomly assigned to either the electrical stimulation (e-stim) group or the sham group to serve as a control. After a two-week habituation period, the mice were subjected to 8 weeks of biweekly e-stim or sham treatments. Twice a week and immediately prior to each e-stim/sham session for the first 4 weeks, mice were injected intraperitoneally with BrdU at a dose of 50 mg/kg in a volume of 10 ml/kg. In cohort 3 only (n = 7 e-stim; n = 8 sham mice), following the 8-week e-stim/sham treatment, mice underwent 5 days of Morris water maze testing, 2 days of rotarod, and 2 days of contextual fear conditioning, in that order, before being perfused with a 4% paraformaldehyde solution 24 h after the final behavioral testing session. These tasks were chosen because mice have previously been shown to display improvements on these tasks after chronic exercise^24^. In cohorts 1 and 2 (n = 10 e-stim mice and 9 sham mice), mice were perfused 24 h after the final e-stim/sham treatment.

### Behavioral testing

Behavioral testing was performed following published procedures from our lab^24^.

#### Morris water maze

A pool with a 40.5-inch diameter was placed in the center of a room with graphically distinct landmarks hanging on each of the 4 walls. A white hanging sheet was used to occlude the experimenter from view during testing. White nontoxic paint was used to ensure that the water in the maze was opaque and the water was monitored regularly to ensure that the temperature always remained around 26 °C ± 1°. Topscan video tracking software was used to track the mouse’s latency (s), distance (mm), and speed (mm/s) to the platform. Each mouse underwent 4 acquisition trials per day for 4 days. Each trial lasted 60 s or until the mouse made it to the platform. To complete a trial, each mouse had to find the 9-cm diameter platform on their own and remain on it for 10 s within the 60-s window. If the mouse did not find the platform on their own or remain on it for 10 s within the 60-s window, they were gently guided to the platform by the experimenter. Immediately after each trial, the mice were removed from the maze and were given 30 s to rest. During these 30 s, the mice were placed into a holding cage under a heat lamp and allowed to air dry. A 24-h probe trial occurred on the 5th day. During the probe trial, the platform was removed and the mice were allowed to explore the water maze for 60 s.

#### Rotarod

Mice underwent 2 days of rotarod testing. Mice were tested 4 at a time and underwent 4 trials per day. During each trial, mice were placed on a rotating dowel that accelerated at a rate of 30 revolutions per minute per minute. Latency to fall off the dowel was recorded. The rotarod was wiped down with clidox between trials.

#### Contextual fear conditioning

On day 1, mice were placed in the contextual fear conditioning chamber. At 120 and 150 s, they received a mild footshock (0.5 mA, 2 s duration, 30 s apart). 30 s after the last shock, animals were returned to their home cages. Between mice, the chamber, grid, and the table under the grid were wiped down with clidox. On the following day, animals were placed back in the chamber for a 3-min test in the absence of footshock. Dependent variables were distance traveled (cm), and duration of freezing (s).

### Tissue collection and processing

Mice were perfused with ice cold saline to clear the blood from the body, followed by a 4% paraformaldehyde solution to fix the tissue. Following perfusion, the brains (all experiments and all cohorts) and muscles (Experiment 3, cohort 2 only, n = 8 e-stim and 8 sham) were dissected. Brains were placed in a 4% paraformaldehyde (T353-500; Thermo Fisher Scientific, Pittsburgh, PA) solution overnight and then were transferred to 30% sucrose following established procedures. Brains were sectioned using a cryostat into 40-micron coronal sections and stored in cryoprotectant at − 20 °C. Gastrocnemius and tibialis anterior muscles were collected, weighed, and immediately frozen in precooled isopentane.

### Immunohistochemistry on brain sections

Immunohistochemistry for cFos, Ki67, BrdU, Dcx, NeuN and S100ß in brain sections was performed following published procedures from our lab^25–27^. For details see Supplementary Methods.

#### Experiment 1

Separate 1-in-6 series of brain sections were processed for cFos (marker of neuronal activation; all cohorts, n = 16 per group) and Ki67 (marker of cell division; cohort 2 only, n = 8 per group) immunohistochemistry with diaminobenzidine (DAB) as the chromogen.

#### Experiment 3

Separate 1-in-6 series were processed for BrdU (all cohorts) and doublecortin (Dcx, immature neuron marker; only cohorts 1 and 2; n = 8 e-stim mice and 11 sham mice) immunohistochemistry using DAB as the chromogen. In addition to the DAB analysis, a few sections from each animal in cohort 3 were used to estimate the proportion of BrdU-positive cells that were differentiated into neurons versus astrocytes by using BrdU/NeuN/S100ß immunofluorescence and confocal microscopy.

### Image analysis of brain sections

Image analysis followed published procedures in our lab^25^.

#### Experiment 1 (cFos-DAB, Ki67-DAB)

The entire bilateral DG represented in the 1-in-6 series throughout the rostro-caudal extent of the hippocampus was imaged using a brightfield light microscope (Zeiss AxioScope A1; Carl Zeiss, Inc., Thornwood, NY) equipped with a Zeiss AxioCam MRc5 digital camera and photographed under 100× total magnification (AxioVision SE64 Rel. 4.8 viewer software). Number of cFos- and Ki67-positive cells were counted manually within the granule and subgranule cell layers of the DG by an investigator blind to the experimental conditions, discounting cells cut in the top plane of the section, to generate unbiased estimates of cell numbers. The area of the granule cell layer was also traced using ImageJ (version 1.46r) to generate an estimate of volume.

#### Experiment 3 (BrdU-DAB, Dcx-DAB)

Sections stained for BrdU-DAB and Dcx-DAB were analyzed the same way as cFos and Ki67 in Experiment 1, except for BrdU, in addition to the DG, the CA1/CA2 subfields of the hippocampus and a region of the cortex above the hippocampus were investigated. For the cortex analysis, a frame (dimensions, 643 by 859 µm) was placed adjacent to the midline, sampling both sides (i.e., bilateral) in all sections containing the hippocampus. ImageJ was used to trace the boundaries of the cortex to generate a measure of density of BrdU cells, but since the cortex was not parcellated, volume and total number of cells were not estimated.

#### Experiment 3 (BrdU, S100β, NeuN triple fluorescent label)

In the granule cell layer of the DG, exactly 927 BrdU-positive cells were analyzed from animals in the e-stim treatment, and 960 BrdU-positive cells were analyzed from animals in the sham treatment. In CA1/CA2, 50 BrdU-positive cells were analyzed from e-stim and 47 from sham. Each BrdU-positive cell was examined at 630× total magnification using a Leica SP8 UV/visible laser confocal microscope to determine whether the cell was co-labeled with NeuN or S100ß. A z-stack of images throughout the depth of the section was taken so that each BrdU-positive cell could be analyzed at a focal point with greatest expression of the BrdU-tagged fluorescent label. These images were imported into ImageJ and the average intensity of each channel, BrdU, NeuN, and S100ß over the area of the cell was recorded. These data were then analyzed using the “choisy/cutoff” package in R to obtain a threshold intensity for deciding whether a BrdU positive cell was also NeuN positive or S100ß positive.

### Capillary density in the gastrocnemius and tibialis anterior muscles

Following published procedures from our lab^28^, muscle complexes were divided at the midline along the axial plane and embedded in Tissue-Tek (Fisher Scientific). Three transverse sections per sample were cut for each histological assessment using a CM3050S cryostat (Lecia, Wezlar, Germany). Sections were placed on frozen microscope slides and stored at − 80 °C before staining with rat monoclonal anti-CD31 (BD, San Diego, CA), a marker for endothelial cells. Samples were co-stained with mouse monoclonal anti-dystrophin (MANDRA1) (Sigma-Aldrich, St. Louis, MO) to outline myofibers and measure skeletal muscle area, necessary for calculation of capillary density. For details on immunohistochemistry, see Supplementary Methods. Adobe Photoshop CS5 was used to analyze 5 digital images (randomly selected) per individual, acquired at 200 × total magnification with a Ziess AxioCam digital camera and Axiovision software (Zeiss, Thornwood, NY). All punctate CD31^+^ capillaries were manually counted per image and normalized to image area (capillary density).

### Statistical analysis

SAS (9.3) and R (3.5.1) were used. *P* < 0.05 was considered statistically significant. Data were considered normally distributed if the absolute value of the skewness was less than 1 and kurtosis less than 2, otherwise a power transform was used to transform data to within these boundaries. Posthoc pair-wise differences between means were evaluated using Tukey tests when a statistical interaction was observed or when a main effect with multiple levels was significant. Number of cells (cFos, BrdU, and Dcx), and volume of brain regions (DG, and CA1/CA2) were analyzed using a linear model with treatment (e-stim vs. control) as the main factor and cohort as a blocking variable or cofactor, to remove variation attributed to cohort. We also considered a model with a treatment-by-cohort interaction term, but the term was never significant and therefore was left out of all the final models; moreover, treatment effects were visibly consistent across cohorts. Proportion of BrdU-positive cells in the DG and CA1/CA2 subfields of the hippocampus differentiated into neurons versus astrocytes was analyzed using a Fisher’s exact test. Acquisition of the Morris water maze and latency to fall from the rotarod were analyzed by repeated measures 2-way ANOVA with day as the within-subjects factor and treatment as the between-subjects factor. The following outcome variables were analyzed using an unpaired t-test comparing e-stim to sham control: total number of Ki67 neurons in the DG (and volume of the DG for Ki67 sections), duration in the target quadrant and number of crossings through the platform during the Morris water maze probe test, duration freezing in the contextual fear conditioning test, and serum lactate. Muscle mass was analyzed using analysis of covariance with body mass as the covariate and treatment as the factor. Muscle capillary density was analyzed by 2-way ANOVA with muscle type (gastrocnemius or tibialis anterior), and treatment as factors. Muscle torque during the e-stim procedure was analyzed using 2-way mixed effects ANOVA with individual entered as a random effect to account for the repeated measures, treatment (pre versus post training) entered as a within-subjects factor, and flexion (concentric versus eccentric) as another within-subjects factor. Data from the first contraction were analyzed separately from the last contraction.

## Results

### No activation of the DG from acute e-stim of muscles

**Figure 1:**
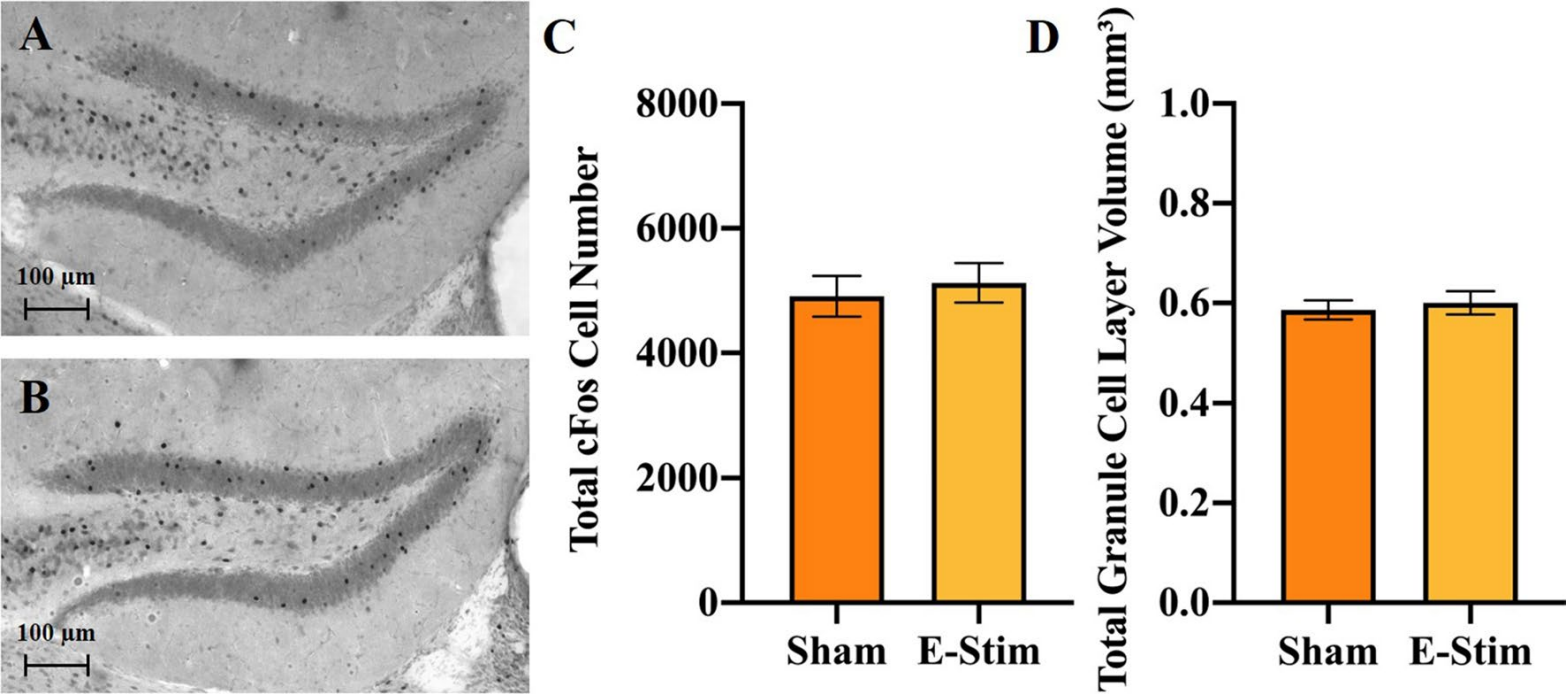
Electrically stimulated muscle contractions do not acutely activate neurons in the DG of the hippocampus. (**A**) and (**B**) Representative sections through the DG stained for cFos from a mouse in the sham and acute e-stim groups, respectively (Experiment 1). (**C**) Mean total number of cFos positive cells in the DG shown separately for sham and e-stim groups. (**D**) Mean volume of the DG. Standard error bars shown; n = 16 per bar.

DG activation was measured in response to the acute e-stim procedure to evaluate the validity of the e-stim model for isolating the contribution of muscle contractions from neuronal activation of the DG, which both typically occur during exercise in awake behaving animals. No significant differences in number of cFos positive cells in the DG (F_1,29_ = 0.22, *P* = 0.64; Fig. 1A–C), volume of the DG (F_1,29_ = 0.27, *P* = 0.61; Fig. 1D) or density (F_1,29_ = 0.02, *P* = 0.89; data not shown) of cFos positive cells were observed. There were significant differences between cohorts for volume of the DG (F_1,29_ = 5.5, *P* = 0.03), probably due to subtle artifactual differences in the intensity of the DAB staining between cohorts, but not cFos number or density of cells.

### Increased serum lactate following acute e-stim of muscles

**Figure 2:**
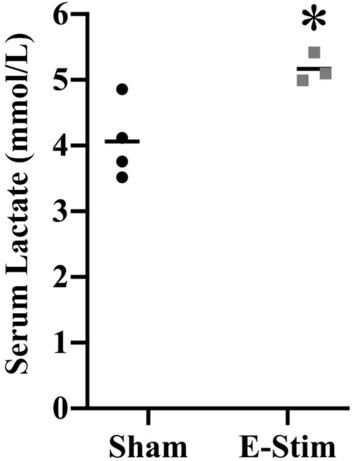
Electrically stimulated muscle contractions acutely increase serum lactate. Individual serum lactate levels shown separately for 4 sham and 3 e-stim mice taken immediately after the e-stim or sham procedure. Horizontal bars show the group mean. *Indicates e-stim group is significantly different from sham (*P* < 0.05).

Serum lactate was measured in response to the acute e-stim treatment to evaluate the validity of the model since increased serum lactate is a typical response when muscle contractions occur during actual exercise in awake behaving rodents^22^. Mice in the e-stim group displayed 27% increased serum lactate relative to sham (t_5_ = 3.1, *P* = 0.02; Fig. 2), typical of what is observed in response to moderate treadmill running in mice^22^.

### Chronic e-stim of muscles increases total number of new cells in the DG

**Figure 3:**
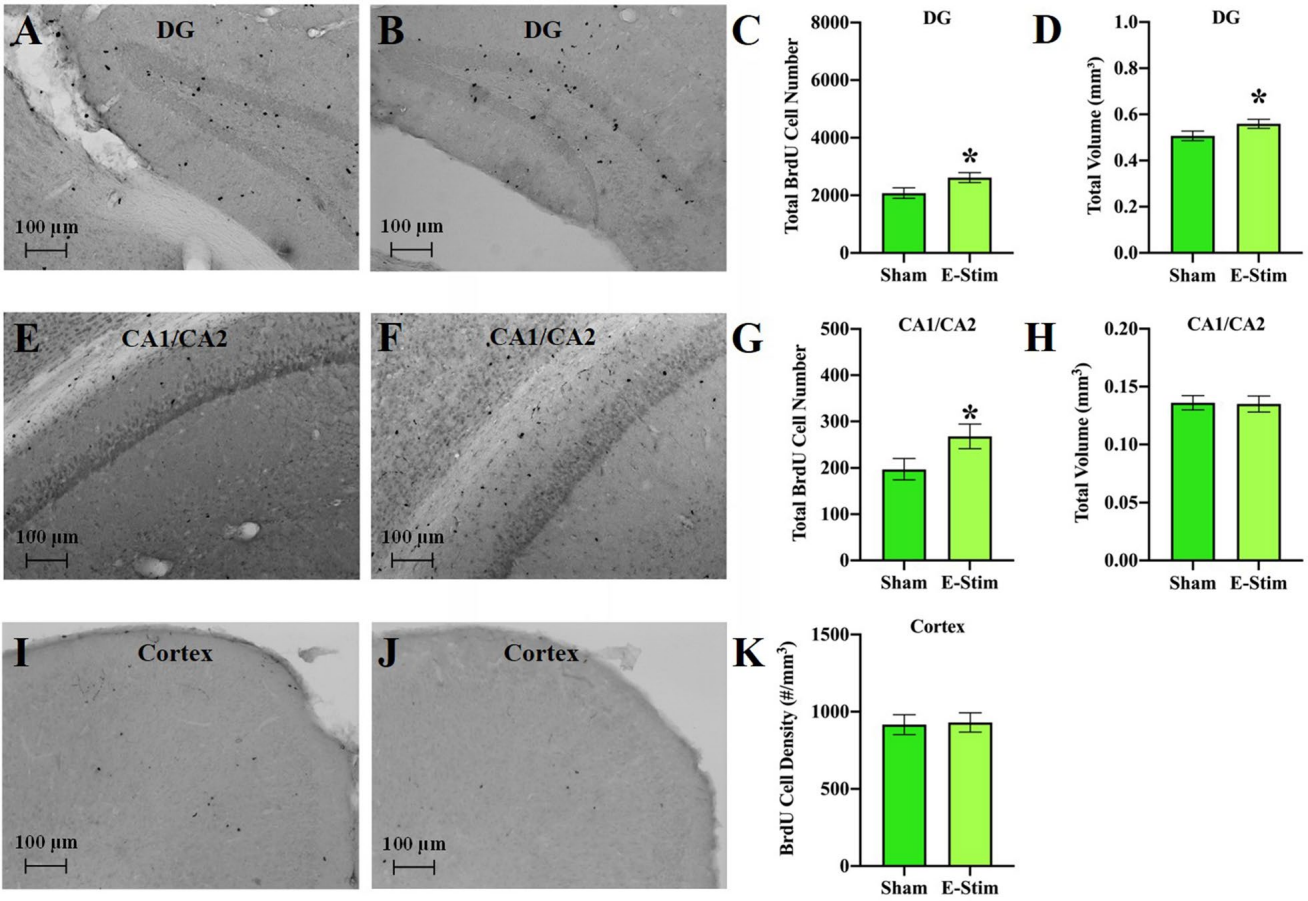
Chronically administered electrically stimulated muscle contractions increase number of new cells in DG and CA1/CA2 subfields of the hippocampus but not in an adjacent region of cortex. (**A**, **B**) Representative sections of the DG stained for BrdU from a mouse in the sham and chronic e-stim groups, respectively. (**C**) Mean total BrdU cell number in the DG shown separately for sham and e-stim groups. (**D**) Mean volume of the DG. (**E**, **F**) Representative sections of the CA1/CA2 region stained for BrdU from a mouse in the sham and chronic e-stim groups, respectively. (**G**) Mean total BrdU cell number in the CA1/CA2 subfields of the hippocampus shown separately for sham and e-stim groups. (**H**) Mean volume of CA1/CA2. (**I**, **J**) Representative sections of the cortex stained for BrdU from a mouse in the sham and chronic e-stim groups, respectively. (**K**) Density of BrdU cells in the cortex. Standard error bars shown; n = 17 per bar. *Indicates e-stim group is significantly different from sham (*P* < 0.05).

To evaluate the impact of chronic muscle contractions alone on cell survival in the DG, we used the BrdU method and analyzed total number of new BrdU cells in the DG, labeled the first 4 weeks of the chronic training period, and subsequently surviving until the 8-week time-point. Mice in the e-stim group displayed 26% greater number of BrdU-positive cells than sham mice in the DG (F_1,30_ = 6.3, *P* = 0.018; Fig. 3A–C), which was associated with a 10% increase in volume of the granule cell layer (F_1,30_ = 4.5, *P* = 0.04; Fig. 3D). No differences in density were observed (F_1,30_ = 2.7, *P* = 0.11; data not shown). There were significant differences between cohorts for total number (F_2,30_ = 28.2, *P* < 0.0001), volume (F_2,30_ = 4.7, *P* = 0.02), and density (F_2,30_ = 45.9, *P* < 0.0001).

### Chronic e-stim of muscles increases astrogliogenesis but not neurogenesis in the DG

**Figure 4:**
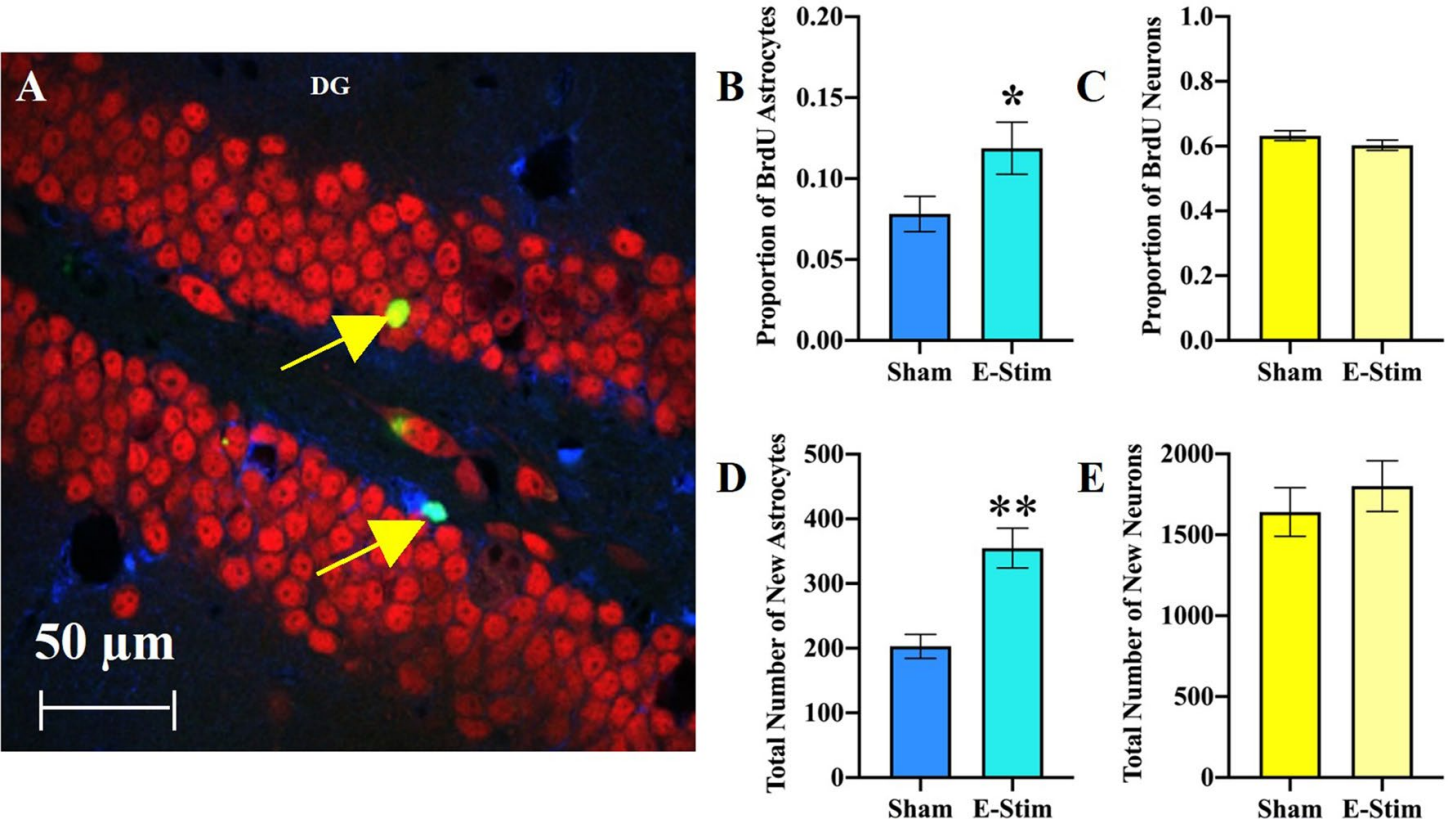
Chronically administered electrically stimulated muscle contractions increase number of new astrocytes but not neurons in the DG. (**A**) Representative section through the DG triple labeled for BrdU (green), NeuN (red), and S100β (blue). The top arrow points to a new neuron, displaying both the green and red label. The bottom arrow points to a new astrocyte, displaying both the green and blue labels. (**B**) Proportion of BrdU cells co-labeled with S100β astrocyte marker in the DG. (**C**) Proportion of BrdU cells co-labeled with NeuN mature neuron marker in the DG. (**D**) Mean total number of new (BrdU-labeled) astrocytes in the DG. (**E**) Mean total number of new (BrdU-labeled) neurons in the DG. Standard error bars shown; n = 17 per bar. * indicates e-stim group is significantly different from sham (*P* < 0.05), ** indicates *P* < 0.0001.

**Figure 5:**
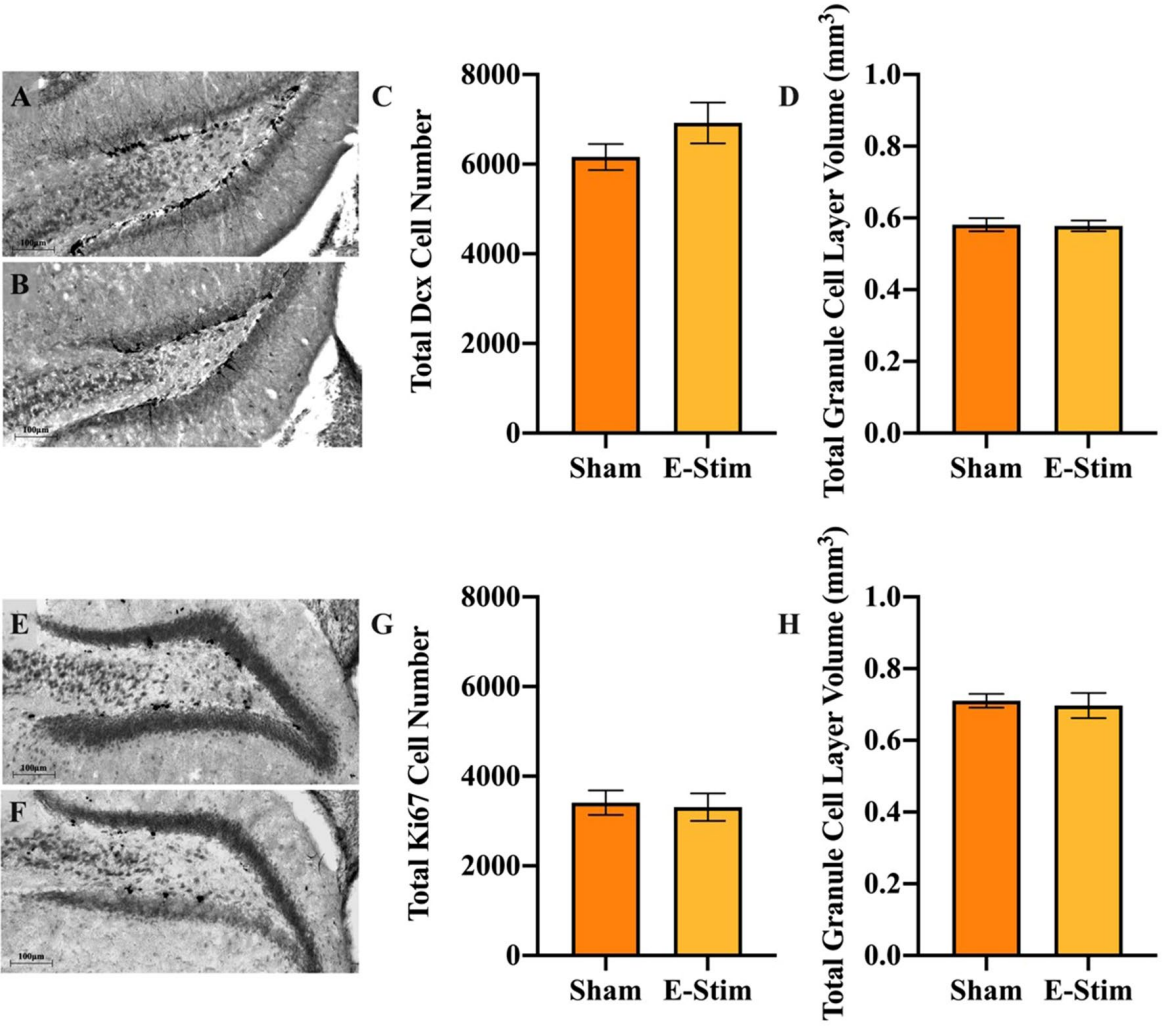
No chronic effect of electrically stimulated muscle contractions on the number of immature neurons or acute effect on cell proliferation in the DG. (**A**, **B**) Representative sections through the DG stained for doublecortin (Dcx), an immature neuronal marker, from a mouse in the chronic sham and e-stim groups, respectively (Experiment 3). (**C**) Mean total number of Dcx positive cells in the DG shown separately for sham and e-stim groups. (**D**) Mean volume of the DG. (**E**, **F**) Representative sections through the DG stained for Ki67, a marker indicating the cell is undergoing mitosis, from a mouse in the acute sham and e-stim groups, respectively (Experiment 1). (**G**) Mean total number of Ki67 positive cells in the DG. (**H**) Mean volume of the DG. Standard error bars shown; n = 16 or 17 per bar.

To identify the phenotype of the increased BrdU cells in DG of the e-stim group, we analyzed a total of 1887 BrdU-positive cells within the DG for co-expression of the astrocyte marker S100β or neuronal marker NeuN to establish the proportion of cells that differentiated into neurons or astrocytes (Fig. 4A). Mice in the e-stim group displayed a greater proportion of BrdU-positive cells differentiated into astrocytes as compared to the sham group (Fisher’s exact test *P* = 0.036; Fig. 4B). No differences in the proportion of BrdU-positive cells differentiated into neurons were observed (*P* = 0.20; Fig. 4C). By multiplying the differentiation rates with total numbers of BrdU-positive cells analyzed above using DAB as the chromogen, we calculated an estimate of the total number of BrdU-positive neurons versus astrocytes in the granule cell layer. Mice in the e-stim treatment displayed 75% increased total numbers of new astrocytes (F_1,30_ = 51.1, *P* < 0.0001; Fig. 4D) but no difference in number of new neurons in the DG (F_1,30_ = 3.6, *P* = 0.07; Fig. 4E). Consistent with this result, no difference in numbers of immature neurons displaying Dcx were observed between groups (F_1,16_ = 0.41, *P* = 0.53; Fig. 5A–D), and there were no significant differences between cohorts (F_1,16_ = 3.8, *P* = 0.07). Acute e-stim did not increase the number of dividing cells expressing Ki67 in the DG of mice from experiment 1 (t_14_ = 0.60, *P* = 0.56; Fig. 5E–H). This suggests that increased astrogliogenesis in the DG from chronic e-stim occurred through increased survival of dividing cells and/or differentiation of progenitor cells toward the astrocyte lineage rather than an acute increase in the proliferation of cells in response to e-stim.

### Specificity of increased astrogliogenesis from e-stim to the hippocampus subfields

To determine the specificity of increased astrogliogenesis to the DG, we examined cell genesis and astrocyte differentiation rates in the CA1/CA2 subfields of the hippocampus. Mice in the e-stim group displayed approximately 50% greater number of BrdU-positive cells than sham mice in the CA1/CA2 subfields (F_1,28_ = 6.3, *P* = 0.02; Fig. 3E–G). No difference in volume was detected (F_1,28_ = 0.07, *P* = 0.79; Fig. 3H), thus density of BrdU cells increased (F_1,28_ = 7.1, *P* = 0.01; data not shown). Cohort was not significant for total number or volume, but was for density (F_2,28_ = 4.7, *P* = 0.02). Approximately 26% of the BrdU cells in the CA1/CA2 fields co-expressed S100β indicating they were astrocytes. No difference in the proportion of new astrocytes was detected between groups (data not shown). None of the BrdU cells expressed NeuN, suggesting there were no new neurons in the CA1/CA2 fields, as expected. Hence, the remaining cells were undifferentiated or differentiated into a different cell type than neurons or astrocytes. No differences between groups in density of BrdU cells were detected in the cortex (F_1,29_ = 0.40, *P* = 0.53; Fig. 3I–K), after correcting for differences between cohorts (F_2,29_ = 22.4, *P* < 0.0001).

### Chronic e-stim of muscles does not improve behavioral performance

To determine the extent to which muscle contractions alone were capable of improving behavioral performance, mice were tested on three tasks known to display improvements from exercise in awake behaving mice: the Morris water maze, contextual fear conditioning and rotarod^24^.

#### Morris water maze

**Figure 6:**
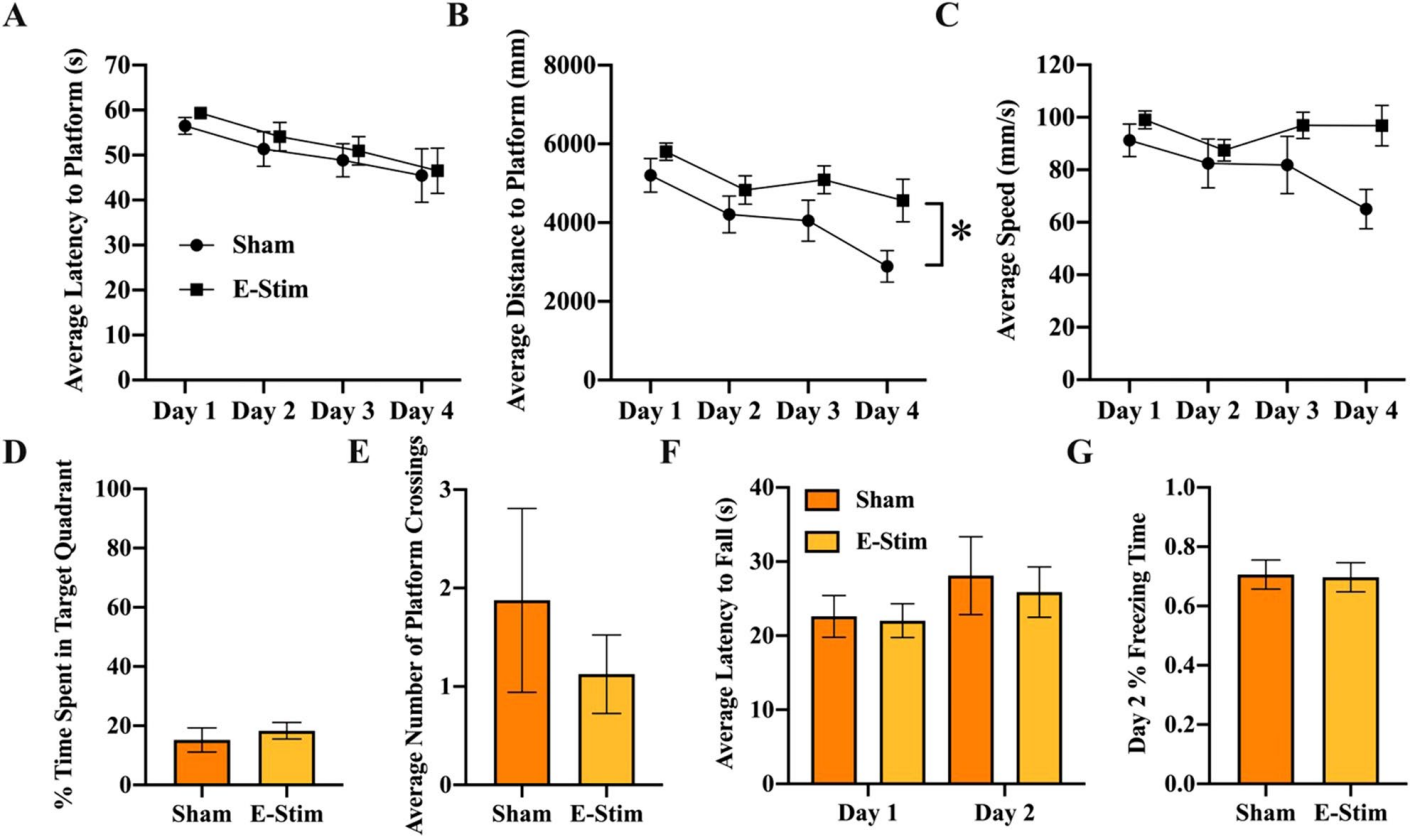
Chronically administered electrically stimulated muscle contractions do not improve behavioral performance. (**A**) Mean latency to reach the platform location in the Morris water maze across the 4 days of acquisition shown separately for sham and e-stim groups. (**B**) Same as A for distance to reach the platform. (**C**) Same as A for swim speed. (**D**) Mean percent duration in the target quadrant during the 1-min probe trial. (**E**) Mean number of crossings through the platform location. (**F**) Mean latency to fall from the rotarod on days 1 and 2 of the test shown separately for sham and e-stim groups. **G** Mean percent duration freezing on day 2 of contextual fear conditioning test. Standard errors shown; n = 8 per bar. *Indicates e-stim group is significantly different from sham (*P* < 0.05).

Mice learned the task as indicated by a significant decrease in both latency (F_3,42_ = 4.37, *P* = 0.009; Fig. 6A) and path length (F_3,42_ = 6.6, *P* = 0.0009; Fig. 6B) to reach the hidden platform over the days. No significant differences in swim speed were detected across days (F_3,42_ = 2.6, *P* = 0.07) or between treatments (F_1,14_ = 3.7, *P* = 0.08; Fig. 6C). Mice in the e-stim group took a slightly longer path to reach the platform collapsed across the days (F_1,14_ = 8.1, *P* = 0.01; Fig. 6B). No treatment differences were observed for latency or speed, and no interactions between day and treatment were detected for any of the variables. No differences in duration in the target quadrant (F_1,14_ = 0.40, *P* = 0.54; Fig. 6D) or number of crossings (F_1,14_ = 0.19, *P* = 0.67; Fig. 6E) through the platform location were detected between groups. Average duration in the target quadrant during the probe test on day 5 was not significantly different from 25% (using 1 sample t-test) for both groups, suggesting the mice did not have a strong memory for where the platform was located 24 h after the last trial on day 4.

#### Rotarod

Average latency (over the 4 trials) to fall from the rotarod was slightly higher, though not significant, on day 2 than on day 1 (F_1,14_ = 4.2, *P* = 0.06; Fig. 6F), but no differences between treatments were detected (F_1,14_ = 0.09, *P* = 0.76). Further, there was no interaction between day and treatment (F_1,14_ = 0.13, *P* = 0.72).

#### Contextual fear conditioning

No differences in duration freezing were detected between treatment groups on day 2 (t_14_ = 0.13, *P* = 0.90; Fig. 6G).

### Chronic e-stim of muscles increases muscle capillary density and muscle endurance

**Figure 7:**
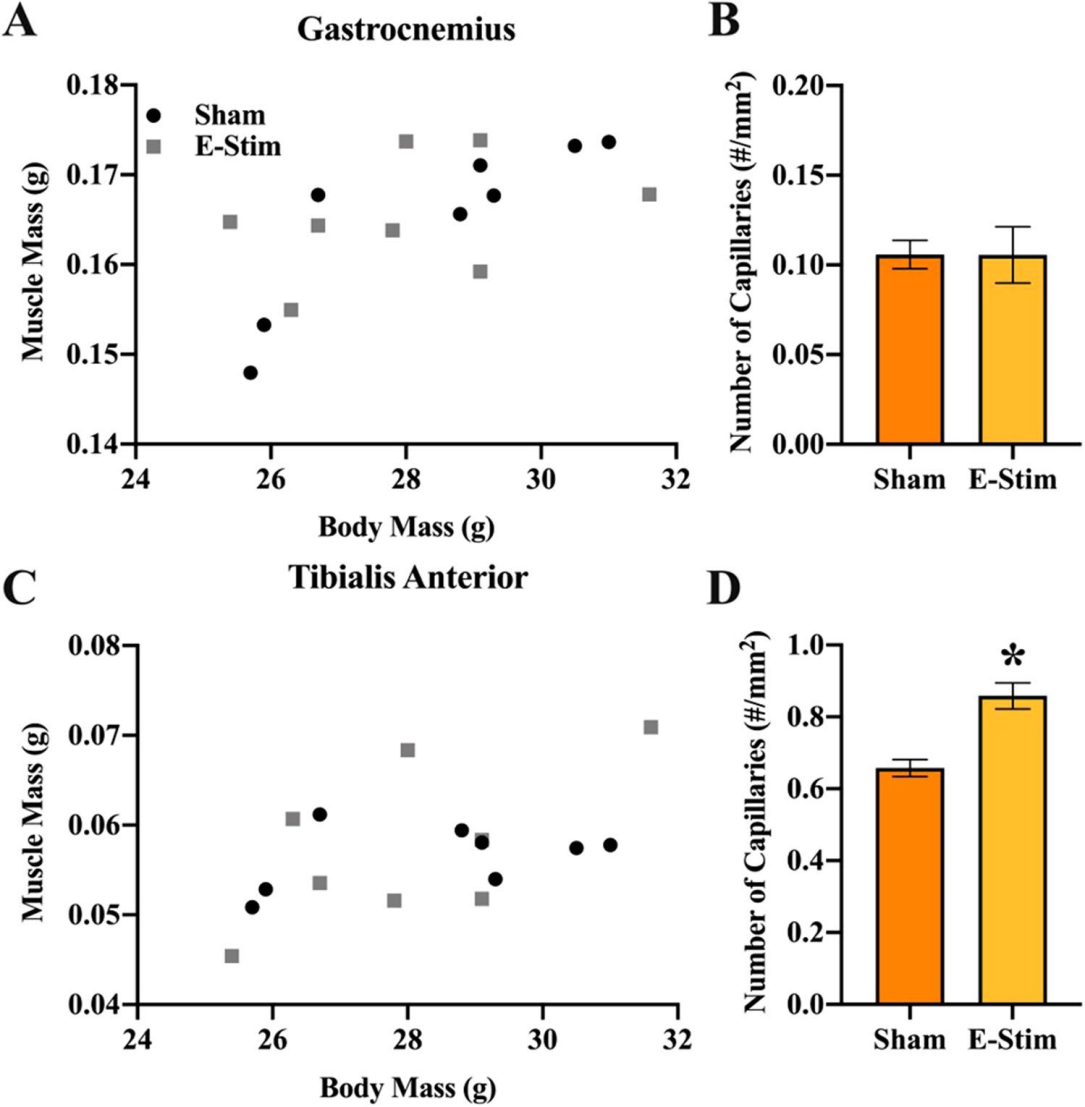
Chronically administered electrically stimulated muscle contractions do not result in muscle hypertrophy but do increase capillary density in the tibialis anterior muscle. (**A**) Mass of the gastrocnemius muscle plotted against body mass across individual mice in the sham and e-stim groups. (**B**) Mean capillary density in the gastrocnemius muscle shown separately for sham and e-stim groups. (**C**) Mass of the tibialis anterior muscle plotted against body mass across individual mice in the sham and e-stim groups. (**D**) Mean capillary density in the tibialis anterior muscle shown separately for sham and e-stim groups. Standard errors shown; n = 8 per bar. *Indicates e-stim group is significantly different from sham (*P* < 0.05).

To confirm that the e-stim procedure produced exercise-physiological adaptations expected of contractions of large muscle masses to the point of fatigue, both the mass, and capillary density of the recruited muscles were evaluated. Masses of both the gastrocnemius (F_1,12_ = 4.9, *P* = 0.048) and tibialis anterior muscles (F_1,12_ = 11.6, *P* = 0.005) were correlated with body mass, but no differences were observed between groups (Fig. 7A,C). Capillary density was approximately sevenfold higher in the tibialis anterior muscle as compared to gastrocnemius complex (F_1,14_ = 857.3, *P* < 0.0001), and there was a significant main effect of e-stim treatment (F_1,14_ = 17.1, *P* = 0.001) and interaction between the muscle type and treatment (F_1,14_ = 20.4, *P* = 0.0005) (Fig. 7B,D). Tukey posthoc tests indicated e-stim had no effect on capillary density in the gastrocnemius complex (*P* = 0.99), but significantly increased capillary density in the tibialis anterior muscle (*P* < 0.0001).

**Figure 8:**
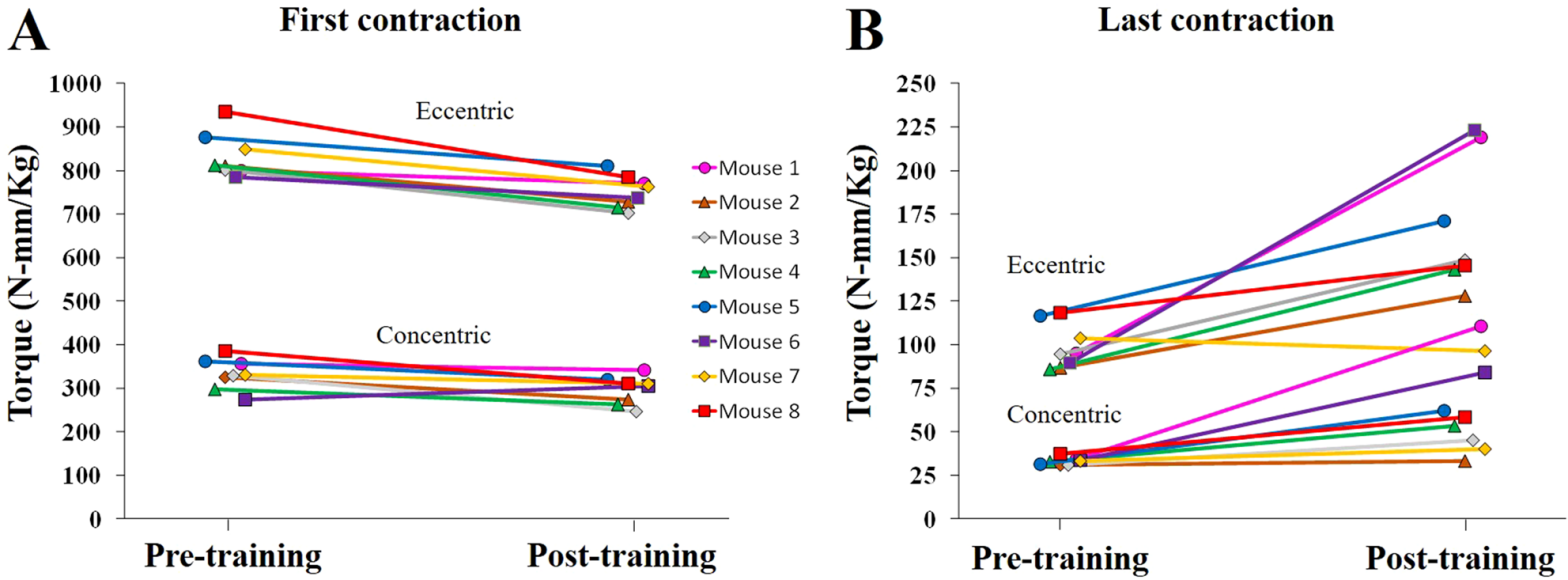
Increased muscle endurance following chronically administered electrically stimulated muscle contractions. (**A**) Torque applied to the foot pedal during the very first electrically stimulated muscle contraction of the session before training (i.e., the very first contraction), and after training, (i.e., the first contraction of the last session after 8 weeks of training). Each individual (Experiment 3, cohort 2) is shown with a separate color line and unique symbol. Torque for eccentric and concentric contractions are shown separately. (**B**) Same as A for the last contraction of the session. Torque significantly increased from pre to post collapsed across eccentric and concentric contractions (*P* < 0.05) indicating increased muscle endurance.

To evaluate the extent to which the chronic e-stim procedure resulted in exercise-training of the muscles, peak torque applied by the muscles on the first day of training was compared to peak torque on the last day. Peak torque at first contraction was greater on the first day of training as compared to after 8 weeks of training for both concentric and eccentric contractions (F_1,21_ = 44.3, *P* < 0.0001; Fig. 8A). Eccentric contractions generated approximately 2.5-fold as much torque as concentric contractions (F_1,21_ = 2,897.9, *P* < 0.0001). There was a significant interaction between flexion type (eccentric versus concentric) and day (pre or post training) (F_1,21_ = 6.9, *P* = 0.02), because the absolute difference between pre and post was greater for eccentric contractions than concentric by virtue of the higher torque.

Peak torque at last contraction was greater on the last day of training as compared to the first indicating muscle endurance (F_1,21_ = 30.1, *P* < 0.0001; Fig. 8B). Eccentric contractions generated approximately 2.75-fold as much torque as concentric contractions (F_1,21_ = 103.9, *P* < 0.0001). No significant interaction between flexion type and day was detected.

## Discussion

In the present study we show for the first time that chronic muscle contractions alone increase astrogliogenesis in the adult hippocampus (Fig. 4D), but are not sufficient to enhance neurogenesis (Fig. 4E) or behavioral performance (Fig. 6). Importantly, the results demonstrate that muscle contractions, in the absence of hippocampal neuronal activation (Fig. 1), only partially recapitulate the full effects of physical exercise on the hippocampus. This further highlights that hippocampal neuronal activation might be important to drive neurogenesis during exercise^3,18^. Whilst our results support a growing body of literature suggesting that muscle-derived factors which cross the blood–brain barrier can influence the morphology and neurophysiology of the hippocampus^9–13,20, 29^, it adds to this literature by showing that the contribution of muscle contractions is specifically related to the survival and differentiation of new astrocytes in the hippocampus. Astrocytes are a key component of the neurovascular unit^30^ which provide energy to neurons in the form of lactate^31^ and regulate the release of factors that influence synaptic function and neurogenesis^32–34^, all of which are upregulated during physical exercise. Perhaps it is not surprising that survival and differentiation of new astrocytes in the hippocampus are sensitive to blood-born factors, given that astrocytes are a key component of the blood–brain barrier and are likely to be one of the first brain cells to detect blood-born factors released from muscle. Conversely, it is intriguing that increased astrogliogenesis induced from muscle contractions was specific to the hippocampus, with no changes detected in the adjacent cortex (Fig. 3K). Given that exercise has been observed to increase the number of astrocytes in the cortex^35^, our results suggest that cortical astrogliogenesis may require neuronal activation or some other component of exercise in addition to muscle contractions. The mechanisms by which myokines may selectively impact the hippocampus is an unexplored area and should be the focus of future research.

The lactate released from muscle measured in this study (Fig. 2), or other specific growth factors and myokines could have contributed to increased astrogliogenesis in the hippocampus. Previous studies have identified factors such as lactate, VEGF, IGF-1, irisin, and cathepsin B, as important to drive increases in BDNF expression and adult hippocampal neurogenesis^9−13^. Astrogliogenesis occurs in proportion to neurogenesis to support the new neurons^32,35–37^. Hence, astrocytes rather than immature developing neurons could have been the direct target of the blood-born muscle factors in the above mentioned studies. More work is needed to characterize the sequelae of chemicals released from contracting muscles in the e-stim model to substantiate this hypothesis. Alternatively, it is possible that stimulation of astrogliogenesis in the hippocampus was induced via peripheral nerves responding to the contractions and signaling to the hippocampus through the somatosensory cortex^20^. However, if this was the case it is interesting that it did not result in increased neuronal activation of the DG as indicated by cFos immunohistochemistry (Fig. 1)^25^. In future studies, it may be possible to sever the sensory information deriving from the peripheral nerves in the hind limbs to test directly the hypothesis that signaling molecules originated in the muscle are driving brain adaptations. Yet, this would undoubtedly cause other problems in a chronic study.

Exercise is widely known to increase numbers of astrocytes in the DG along with and in proportion to the numbers of new neurons, and micro blood vessels^5,36^. This is demonstrated by a large increase in numbers of BrdU cells in the DG in response to exercise training (from 2 to 5-fold depending on the mouse strain), a small percentage of which (less than 10%) are astrocytes^4,26,37–39^. The niche created by the local astrocytes in the DG is critical for progenitor cell differentiation towards the neuronal lineage^34^. Interestingly, in the present study we observe an increase in new astrocytes but not new neurons. One important feature that was not observed during muscle contraction was neuronal activation of the DG, which is typically present during regular physical exercise in an awake and behaving animal (Fig. 1)^25,40^. It is possible that the combination of neuronal activation of the DG and muscle contraction during actual physical exercise is required to produce the total neurogenic response^3^. Alternatively, there is recent evidence to suggest that systemic levels of Gpld1, a glycosylphosphatidylinositol (GPI)-specific phospholipase D1, produced by the liver during exercise, might also play an important role in exercise-induced neurogenesis and improved cognitive function in aged rodents^41^. The absence of increased neurogenesis may partially explain the lack of cognitive improvements in contextual fear conditioning (Fig. 6G), and significantly impaired performance of e-stim relative to sham during acquisition of the Morris water maze (Fig. 6B). The uncoupling of the muscle contractions from hippocampal neuronal activity, which typically would go together in an exercising awake behaving mouse, may have resulted in a mismatch between astrogliogenesis and neurogenesis, resulting in slightly impaired function of the hippocampus. An alternative possibility is that the impaired performance is a result of elevated stress hormones and/or inflammation resulting from the chronic e-stim treatment. Though we did not measure stress hormones or inflammation markers in this study, an extensive literature shows that these factors decrease adult hippocampal neurogenesis which was not observed here (Fig. 4E)^42–45^, thus minimizing this possibility.

The 10% increase in volume observed in the DG from the e-stim treatment (Fig. 3D) is similar to what occurs in response to voluntary wheel running exercise^26^. However, increased neurogenesis is established to contribute to the volume expansion in the voluntary wheel running paradigm, and the e-stim treatment did not increase neurogenesis. It is possible that increased astrogliogenesis (Fig. 4D) contributed to the volume difference (Fig. 3D), but may not have been the only factor. The possibility that muscle contractions alone might increase angiogenesis, myelination, or enlargement of the neuron cell bodies in the DG needs to be explored in future studies.

The important feature of the e-stim model for the purpose of this study was to induce repeated contractions of muscles to the point of fatigue, without inducing neuronal activation of the hippocampus that would otherwise occur in an awake exercising animal^3^. Importantly, the e-stim treatment did not result in DG neuronal activation (Fig. 1), which normally occurs in awake rodents as an acute response whenever they are exercising, in proportion to exercise intensity^25,46–50^. The lack of DG activation allowed for an accurate isolation of the muscle contribution to exercise-induced neuroadaptations. The hind limb muscles were contracted to the point of fatigue over the course of the session as indicated by the order of magnitude reduction in torque for the last contraction relative to the first (compare y-axis range between panel A and B in Fig. 8). The reason why training decreased torque for the first contraction is not clear; it could be related to a physiological tradeoff between power of the muscle contraction and endurance. Chronically, the e-stim model resulted in increased capillary density in the tibialis anterior (Fig. 7D), along with increased torque from the last contraction after training (Fig. 8B) and no muscle hypertrophy (Fig. 7A,C), which collectively demonstrates that muscles were physiologically adapted to training in a way that supported endurance^51^. However, it is important to note that the muscle contractions induced from the e-stim treatment do not necessarily represent how muscles would contract during actual exercise awake behaving mice. The contractions are likely much stronger from e-stim than mice would normally be able to perform, voluntarily. The sciatic nerve was electrically activated which contracts the entire muscle. Normally only a fraction of the nerves would be recruited in a spatially and temporally different pattern, which would be exceedingly difficult to mimic in its entirety. In the future, it would be useful to establish the physiological similarities and differences between our e-stim and other forms of real exercise to better characterize the e-stim model.

One potential limitation of the present study is that all the mice were anesthetized with isoflurane which could have had an impact on the outcome measures, e.g., neurogenesis, astrogliogenesis, or behavioral performance. The anesthesia was, of course, necessary to perform the electrical stimulation procedure, and since all the mice received a similar dose of isoflurane, it cannot explain the differences we detected between groups in neuroanatomy of the hippocampus (Figs. 3 and 4) and behavioral performance measures (Fig. 6B). Moreover, although there is some evidence that isoflurane can reduce adult hippocampal neurogenesis when administered in early postnatal period^52^, it does not affect neurogenesis in adults^53^. Finally, the levels of neurogenesis that we observed (Fig. 4E) is comparable to what we and others have observed in naïve 3-mon old, sedentary, male, C57BL/6 J mice^26,54^.

In summary, our study is the first to isolate the contribution of muscle contractions to the long-term impacts of exercise on neuroanatomy. We discovered that while muscle contractions alone are sufficient to increase the number of astrocytes specifically in the hippocampus, they were insufficient to increase neurogenesis or improve behavioral performance, as typically observed during physical exercise. We conclude that muscle contractions only contribute partially to exercise-induced brain adaptations and suggest that neuronal activation of the hippocampus is likely required to increase adult hippocampal neurogenesis and improve behavioral performance.

## Supplementary information

Supplementary Information
