# Supplementary material for: Electrically stimulated hind limb muscle contractions increase adult hippocampal astrogliogenesis but not neurogenesis or behavioral performance in male C57BL/6J mice
Source: Sci Rep. 2020 Nov 9;10:19319. doi: 10.1038/s41598-020-76356-z (PMC7652861; doi:10.1038/s41598-020-76356-z)
Supplement: Supplementary file 1 — Supplementary Information [file 41598_2020_76356_MOESM1_ESM.docx]

**Electrically stimulated hind limb muscle contractions increase adult hippocampal astrogliogenesis but not neurogenesis or behavioral performance in male C57BL/6J mice**

Jennie C. Gardner, Svyatoslav V. Dvoretskiy, Yanyu Yang, Sanjana Venkataraman, Dominica A. Lange, Shiping Li, Alexandria L. Boppart, Noah Kim, Catarina Rendeiro, Marni D. Boppart, & Justin S. Rhodes

**Supplementary Methods**

**Experiment 1: cFos immunohistochemistry (DAB as the chromogen):** Sections were washed 4 times for 5 minutes in 0.02M PBS before being treated in a 0.6% H_2_O_2_ (H325-500; Thermo Fisher Scientific, Pittsburgh, PA) solution for 20 minutes. Sections were then washed 3 times for 5 minutes in PBS, blocked in PBS-X (0.2% Triton X-100; Sigma Aldrich, St. Louis, MO) with 6% normal goat serum (NGS) (S26; EMD Millipore Corporation, Billerica, MA) for 1 hour, and incubated with an anti-cFos primary antibody (AB-190289, 1:10,000; Abcam, Cambridge, MA) in PBS-X with 3% NGS at 4°C for 48 hours. On day 2, sections were washed 4 times for 5 minutes in PBS-X with 3% NGS before being incubated in a biotinylated goat anti-rabbit secondary antibody (BA-1000, 1:200; Vector Labs, Burlingame, CA) for 90 minutes at room temperature. Sections were then washed in PBS-X 4 times for 5 minutes and incubated in an avitin-biotin complex (PK-6100 VECTASTAIN Elite ABC kit; Vector Labs, Burlingame, CA) for 1 hour. Sections were washed 4 times for 5 minutes in PBS-X again, incubated with DAB, and washed 4 times for 5 minutes in PBS. Tissue was left in PBS at 4°C overnight before being mounted on gel-subbed microscope slides. After the slides were completely dry, they were dehydrated and stained with methylene blue (2 min. 70% EtOH, 2 min. 50% EtOH, 5 min. 0.2M PB, 5 min. periodic acid, 6 minutes methylene blue, 5 minutes 0.2M PB, 2 min. 50% EtOH, 30 sec. 70% EtOH, 2.5 min. 95% EtOH, several dips 100% EtOH, several dips 100% EtOH, 10 min. xylenes) and coverslipped (2975-246, Corning Cover Glass, 24x60 mm; Corning Life Sciences, Nuevo León, Mexico) using Permount (SP15-500; Thermo Fisher Scientific, Pittsburgh, PA).

**Experiment 1: Ki67 immunohistochemistry (DAB as the chromogen):** Sections were washed 3 times for 5 minutes in TBS before being treated in a 0.6% H_2_O_2_ (H325-500; Thermo Fisher Scientific, Pittsburgh, PA) solution for 30 minutes. Sections were then washed 3 times for 5 minutes in TBS, blocked in TBS-X (0.3% Triton X-100; Sigma Aldrich, St. Louis, MO) with 3% normal goat serum (NGS) (S26; EMD Millipore Corporation, Billerica, MA) for 30 minutes, and incubated with an anti-Ki67 primary antibody in rabbit (AB-15580, 1:500; Abcam, Cambridge, MA) in TBS-X with 3% NGS at 4°C for 72 hours. On day 2, sections were washed 3 times for 5 minutes in TBS and rinsed with TBS-X plus 3% NGS before being incubated in a biotinylated goat anti-rabbit secondary antibody (BA-1000, 1:250; Vector Labs, Burlingame, CA) for 100 minutes at room temperature. Sections were then washed in TBS-X 3 times for 5 minutes and incubated in an avitin-biotin complex (PK-6100 VECTASTAIN Elite ABC kit; Vector Labs, Burlingame, CA) for 1 hour. Sections were washed 4 times for 5 minutes in TBS-X again, incubated with DAB for 5 minutes, and quickly washed 2 times with TBS. Tissue was then immediately mounted on gel-subbed slides and allowed to dry completely before being dehydrated, stained with methylene blue, and coverslipped using Permount as described above.

**Experiment 3: BrdU immunohistochemistry (DAB as the chromogen):** Sections were washed 4 times for 5 minutes in TBS before being treated in a 0.6% H_2_O_2_ (H325-500; Thermo Fisher Scientific, Waltham, MA) solution for 30 minutes. Sections were then washed 3 times for 5 minutes in TBS and treated with a 50% de-ionized formamide (AB00600-00500; American Bio, Canton, MA) 10% 20xSSC buffer (BP1325-1; Thermo Fisher Scientific, Pittsburgh, PA) solution at 65°C for 90 minutes. Sections were then rinsed with 2xSSC buffer for 15 minutes and treated with 2N HCl (A144C-212; Thermo Fisher Scientific, Pittsburgh, PA) for 30 minutes at 37°C. Tissue was treated with borate buffer (0.1 M, pH 8.5) for 10 minutes at room temperature and washed 3 times for 5 minutes in TBS. Finally, sections were treated with TBS-X (0.1% Triton X-100) with 3% NGS for 30 minutes and incubated with an anti-BrdU primary antibody (AB-6326, 1:200; Abcam, Cambridge, MA) in TBS-X plus NGS at 4°C for 72 hours. After the 72-hour incubation period, tissue was rinsed in TBS 4 times for 5 minutes and rinsed in TBS-X plus 3% NGS for 30 minutes. Tissue was then incubated in a biotinylated goat anti-rat secondary antibody (BA-9400, 1:250; Vector Labs, Burlingame, CA) for 100 minutes at room temperature. Sections were washed in TBS-X 4 times for 5 minutes, incubated in DAB for 5 minutes, quickly rinsed 2 times in TBS, and then washed in TBS 3 times for 5 minutes. Tissue was then immediately mounted on gel-subbed slides and allowed to dry completely before being dehydrated, stained with methylene blue, and coverslipped using Permount as described above.

**Experiment 3: Dcx immunohistochemistry (DAB as the chromogen):** Sections were washed 3 times for 5 minutes in 0.02M PBS before being treated in a 3% H_2_O_2_ solution in PBS-X (H325-500; Thermo Fisher Scientific, Pittsburgh, PA) for 30 minutes. Sections were then washed 3 times for 5 minutes in PBS, blocked in PBS-X (0.3% Triton X-100; Sigma Aldrich, St. Louis, MO) with 3% normal goat serum (NGS) (S26; EMD Millipore Corporation, Billerica, MA) for 30 minutes, and incubated with an anti-Dcx primary antibody in rabbit (4604S, 1:1000; Cell Signaling Technology, Danvers, MA) in PBS-X with 3% NGS at 4°C for at least 16 hours. On day 2, sections were washed 3 times for 5 minutes in PBS before being incubated in a biotinylated goat anti-rabbit secondary antibody (BA-1000, 1:500; Vector Labs, Burlingame, CA) in PBS for 2 hours at room temperature. Sections were then incubated in an avitin-biotin complex (PK-6100 VECTASTAIN Elite ABC kit; Vector Labs, Burlingame, CA) for 1 hour. Sections were washed in PBS 3 times for 5 minutes, incubated with DAB for 5 minutes, and washed 3 times for 5 minutes in PBS. Tissue was then immediately mounted on gel-subbed slides and allowed to dry completely before being dehydrated, stained with methylene blue, and coverslipped using Permount as described above.

**Experiment 3: Phenotyping BrdU cells through triple labeling:** A similar procedure as described above for BrdU immunohistochemistry was used except a cocktail was used for the primary antibody step consisting of an anti-BrdU primary antibody from rat (AB-6326, 1:100; Abcam, Cambridge, MA), an anti-NeuN primary antibody from rabbit (AB-177487, 1:250; Abcam, Cambridge, MA), and an anti-S100ß primary antibody from chicken (287-006, 1:1000; Synaptic Systems, Göttingen, Germany). The secondary antibodies were also delivered in a cocktail consisting of a goat anti-chicken secondary antibody (DyLight 405, 1:250; Jackson Immuno Research Laboratories, West Grove, PA), a goat anti-rabbit secondary antibody (Alexa Fluor 647, 1:250; Jackson Immuno Research Laboratories, West Grove, PA), and a goat anti-rat secondary antibody (Alexa Fluor 488, 1:250; Jackson Immuno Research Laboratories, West Grove, PA). After the secondary step, sections were washed as described above and immediately mounted on gel-subbed slides, and coverslipped using ProLong Gold antifade reagent (P36930; Thermo Fisher Scientific, Pittsburgh, PA). Slides were stored at 4°C prior to microscopy.

**Experiment 3: Immunohistochemistry for detecting capillary density in the muscles.**

Frozen sections were fixed in ice-cold acetone for 10 min and blocked with PBS containing 5% bovine serum albumin (BSA) and 70 µg/ml AffiniPure anti-mouse fab fragments diluted in 5% BSA (Jackson ImmunoResearch, West Grove, PA) for 1 hour. Both primary antibodies were diluted to a concentration of 1:100 in PBS with 1% BSA and were applied to the tissue sections for 60 min at room temperature. Fluorescein isothiocyanate (FITC)-labeled donkey antirat(1:200) and tetramethyl rhodamine isothiocyanate (TRITC)-labeled goat anti mouse (1:100) secondary antibodies (Jackson ImmunoResearch) were used to detect the CD31 and dystrophin antibodies, respectively.
